# Supplementary material for: Red blood cell mannoses as phagocytic ligands mediating both sickle cell anaemia and malaria resistance
Source: Nat Commun. 2021 Mar 19;12:1792. doi: 10.1038/s41467-021-21814-z (PMC7979802; doi:10.1038/s41467-021-21814-z)
Supplement: Supplementary file 1 — Supplementary Information [file 41467_2021_21814_MOESM1_ESM.pdf]

Cao et al: Supplementary Tables and Figures

Supplementary Table 1: Exclusion of possible lectin binding artefacts for HbSS RBCs.

Potential artefact	Exclusion
Binding artefact	
Non-specific binding of GNA lectin	• Wide lectin panel show specificity (Figure 1A)<br>• NPL highly correlates with GNA across all samples (Supplementary Fig. 1A)<br>• Mannan blockade (Supplementary Fig. 1B)<br>• Wide non-SCD panel as well as HbAA, HbAS show negative binding (Supplementary Fig. 1C)
Non-specific binding of sickle cells	Little binding with Annexin V (Supplementary Fig. 1D, E)
Sickle cells permeable to lectins	Lack of binding with, intracellular antibodies, BRIC-132/163 (Supplementary Fig. 1F)
O-GlcNAc detection instead of high mannose	No surface binding to RL2 antibody, specific for O-GlcNAc. (Supplementary Fig. 1G for oxidized RBC and Supplementary Fig. 1H for HbSS RBC)
Phenotype artefact	
Reticulocytosis	Lack of expression on non-SCD RBCs showing reticulocytosis (Supplementary Fig. 1I, J).
Sickling process	Morphological independence by microscopy and flow cytometry (Fig. 3A)
Intravascular haemolysis	LDH independence (Fig. 2D-F)

Supplementary Table 2. Proteomic LC-MS analysis of 260kDa band cut from SDS-PAGE of ghosts from healthy (HbAA) RBCs. Proteins included are with minimum: 99.5% probability, 3 peptides identified, and 5.0% sequence coverage. Common contaminants (e.g. keratins and trypsin) have been removed. Identified proteins (UniProt accession, entry codes and description) were sorted based on the number of sequence coverage (%Cov). MW, molecular weight in Da; PLGS score, ProteinLynx Globar Server score.

**Accession**	**Entry**	**Description**	**MW**	**PLGS score**	**Prob. (%)**	**Peptides**	**%Cov**
P02549	SPTA1_HUMAN	Spectrin alpha chain, erythrocytic 1 OS=Homo sapiens	279840	9.9	100.0	125	53.5
P11277	SPTB1_HUMAN	Spectrin beta chain, erythrocytic OS=Homo sapiens	246313	9.9	100.0	46	26.6
P02730	B3AT_HUMAN	Band 3 anion transport protein OS=Homo sapiens	101727	9.9	100.0	14	19.0
P16157	ANK1_HUMAN	Ankyrin-1 OS=Homo sapiens	206136	9.9	100.0	25	17.0
P11166	GTR1_HUMAN	Solute carrier family 2, facilitated glucose transporter member 1 OS=Homo sapiens	54048	9.9	100.0	3	6.3

Supplementary Table 3. Proteomic analysis of F40 trypsin resistant gel band identified α-spectrin peptides (accession P02549, entry SPTA1_HUMAN), sorted on intensities. Peak MW, molecular weight of the protonated (MH+) peptide found in Da; peptide MW, theoretical molecular weight of the protonated (MH+) peptide in Da; delta, difference between peak MW and peptide MW in ppm; score, PLGS score. In peptide sequence, cysteine (C) amino acids in **bold** and underlined correspond to carboxymethylated cysteine amino acid.

Peak MW (MH+)	Peptide MW (MH+)	Delta (ppm)	Score	Sequence		Sequence	Intensity
				Start	End		
1550.795	1550.795	0.08	6.8	280	292	IKEKEPVLTSEDY	317025
1135.598	1135.599	-1.18	6.6	293	303	GKDLVASEGLF	226142
643.359	643.356	4.36	6.3	54	58	HLQVF	146116
1245.657	1245.659	-1.14	6.1	270	279	KRDVTEAIQW	119871
1203.611	1203.612	-0.93	5.6	59	68	KRDADDLGKW	34163
1164.551	1164.553	-2.02	5.5	82	91	EDPTNIQGKY	63659
1263.555	1263.556	-0.65	5.4	164	173	VQE**C**ADILEW	66792
852.448	852.446	1.73	5.2	364	370	EKLQATY	23647
966.491	966.489	2.03	5.1	2362	2370	QALAEGKSY	1717
5788.926	5788.981	-9.48	4.8	1869	1920	AVHETRVQNV**C**AQGEDILNKVLQEESQNKEISSKIEALNEKTPSLAKAIAAW	3774
802.330	802.337	-8.22	4.8	2413	2419	TNSYFGN	5889
1603.711	1603.739	-17.40	4.8	902	914	QQYLADLHEAETW	6290
2193.952	2193.977	-11.35	4.8	466	481	DERHRQYEQ**C**LDFHLF	1892
2065.934	2066.004	-33.71	4.7	1944	1963	IADKETSLKTNGNGADLGDF	6335
950.372	950.392	-21.38	4.6	1090	1097	EAGDMLEW	1078
3284.517	3284.539	-6.43	4.5	1385	1410	EKRKKILDQ**C**LELQMFQGN**C**DQVESW	2553
2727.342	2727.442	-36.73	4.4	1124	1146	QKDLNTNEPRLRDINKVADDLLF	1109

Supplementary Fig. 1

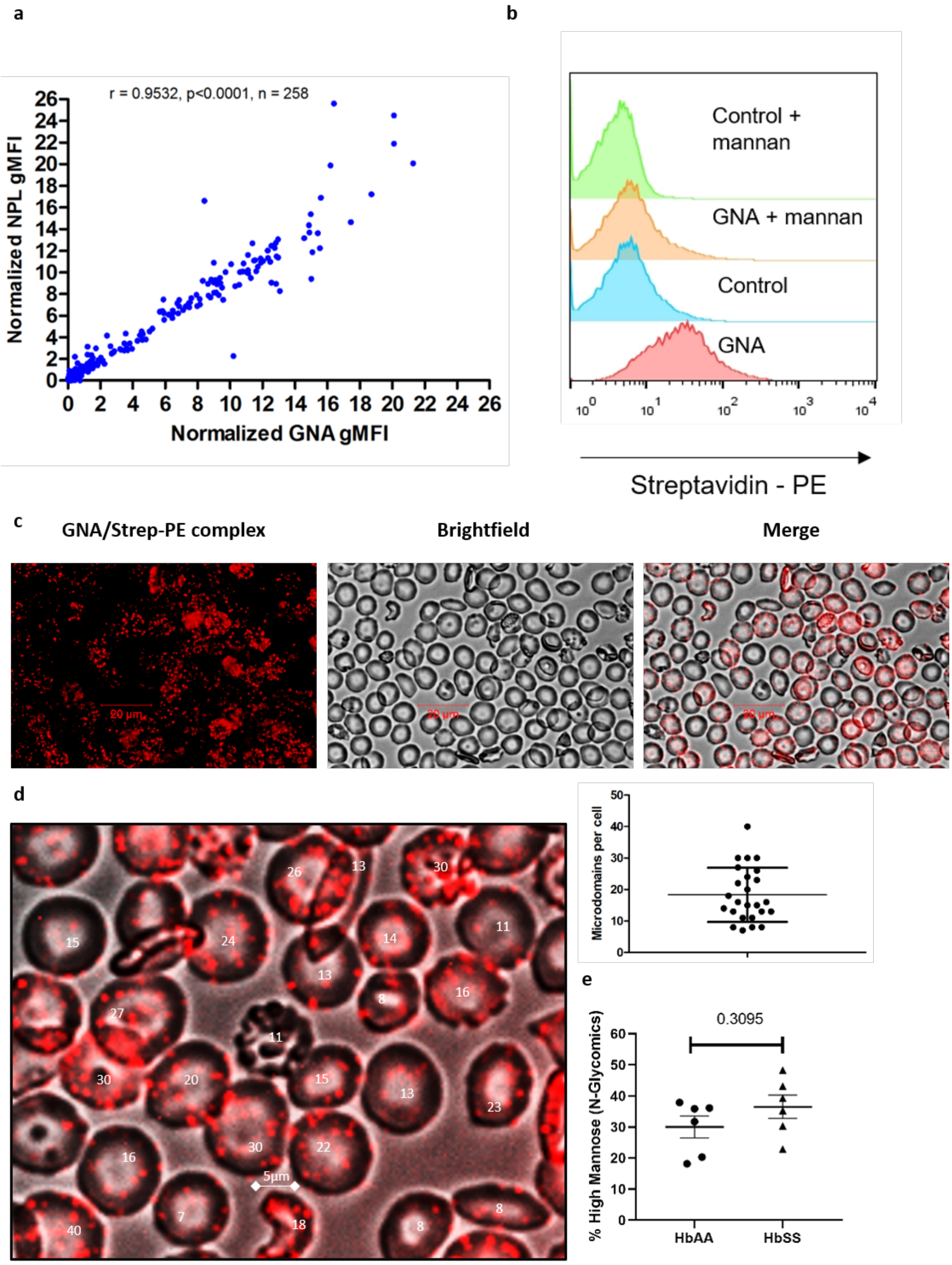

Supplementary Figure 1: Specificity of binding of mannose binding lectins to RBCs.

a) Correlation of *Narcissus pseudonarcissus* Lectin (NPL) with *Galanthus nivalis* Agglutinin (GNA) lectin surface binding; normalized geometric mean fluorescence (gMFI), Spearman's rank correlation. $p=5.5\times10^{-135}$, n=256, independent lectin measurement over more than ten independent experiments.

b) Flow cytometric histograms of GNA lectin and streptavidin control binding for sickle cell homozygote (HbSS) RBCs with and without mannan blockade. Representative of three independent experiments from different donors.

c) GNA lectin/Streptavidin phycoerythrin (PE) staining of HbSS RBCs is visualized by fluorescence microscopy: PE alone, Brightfield alone and merge.

d) HbSS RBCs from a section of image from c) are counted for the number of GNA lectin binding patches visualized by fluorescence microscopy (left) and plotted on the right. Data shown as mean (18.3) +/- SD (8.6), n=25 independent microdomains, representative of three independent experiments.

e) Percentage high mannose structures, with respect to total N-glycans. Untreated HbAA (n= 2 independent donors) and HbSS (n=5 independent donors) ghosts are analysed by N-glycome mass spectrometry. Results are pooled from four independent experiments. High mannose and complex N-glycans total 100%. Data shown as median +/- IQR, 2 tailed Mann-Whitney statistical test.

Supplementary Fig. 2

a

Non-SCD haemolytic anaemia	Median Normalized GNA gMFI	Significance relative to HbAA	*n*
Beta Thalassaemia	0.3	n.s.	5
HS / HE	1.57	n.s.	6
PNH	0.475	n.s.	16
AIHA	0.43	n.s.	3
HbH	0.21		1
PK deficiency	1.21		1

b

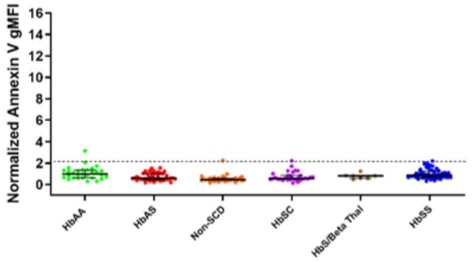

c

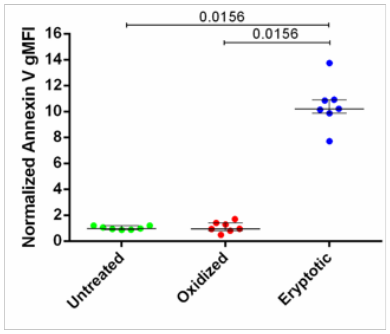

d

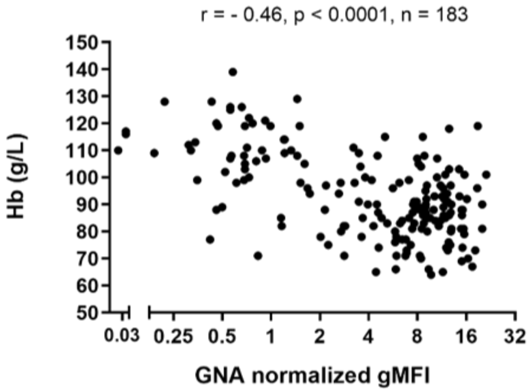

e

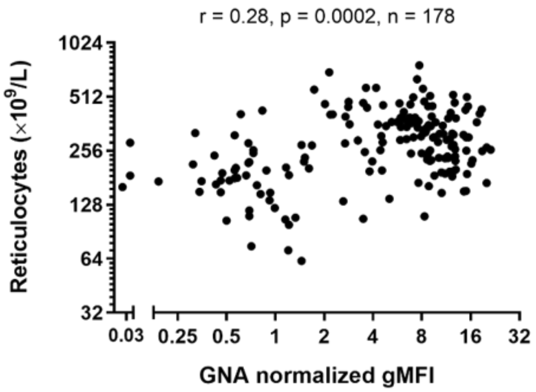

Supplementary Figure 2: Binding of mannose binding lectins and annexin V to RBCs.

a) Table of normalized geometric mean fluorescence (gMFI) values for *Galanthus nivalis* Agglutinin (GNA) lectin binding to non-sickle cell disease haemolytic anaemias, 2 tailed Mann-Whitney tests relative to healthy (HbAA) RBCs. HS/HE (hereditary spherocytosis, hereditary elliptocytosis), PNH (paroxysmal nocturnal haemogobinuria); AIHA (autoimmune haemolytic anaemia); HbH (haemoglobin H disease); PK deficiency (pyruvate kinase deficiency).

b) Normalized gMFI of annexin V binding to RBCs in whole blood samples. Dotted line shows 90th centile of annexin V staining on healthy (HbAA) cells. Non- sickle cell disease anaemias include haemolytic conditions listed in Supplementary Fig. 2a. HbAA (n=29) sickle cell trait (HbAS)(n=42), non- sickle cell disease (n=33), compound heterozygotes for haemoglobins S and C (HbSC)(n=30), compound heterozygotes for haemoglobin S and β-thalassaemia (HbS/Beta Thal) (n=7), homozygotes for sickle cell haemoglobin (HbSS) (n=52). No significant differences by 2 tailed Mann-Whitney. Data shown as median +/- IQR. Each data point indicates an independent RBC donor. Numerous (>10) experiments.

c) Normalized gMFI of annexin V binding to oxidized (copper sulphate/ascorbic acid), eryptotic (calcium ionophore) or untreated purified HbAA RBCs; 2 tailed Wilcoxon. Data shown as median +/- IQR. Single experiment.

d) Plots of haemoglobin concentrations, Spearman's rank, $p=5.8\times10^{11}$,

e) and reticulocyte counts against normalized GNA lectin gMFI for sickle cell disease including HbS/B+, HbSC (compound heterozygosity for HbS with β-thalassaemia and HbC respectively) and HbSS patients. Spearman's rank correlation shown (>20 different experiments).

Supplementary Fig. 3

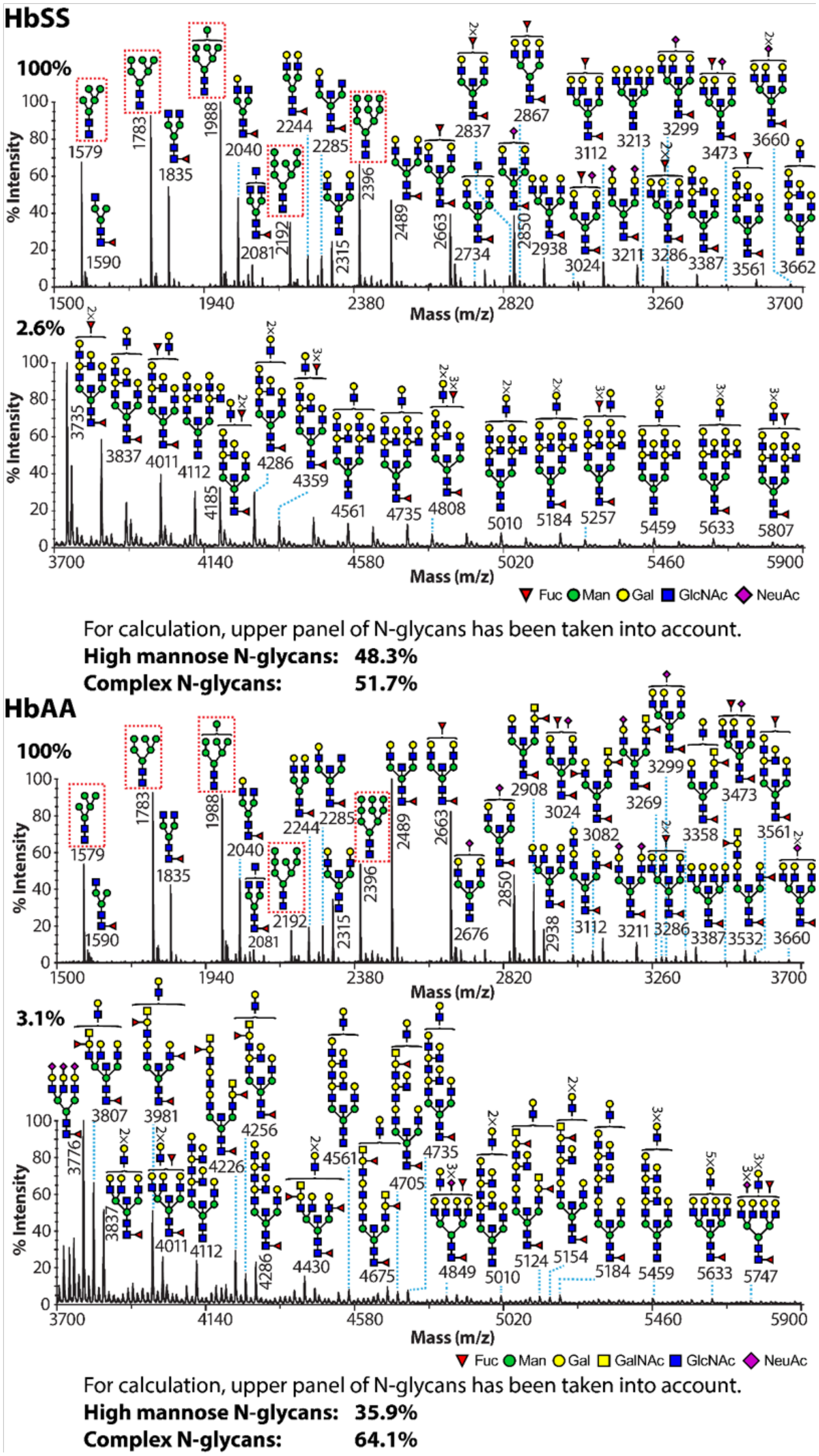

Supplementary Figure 3. Full N-glycome spectra from HbSS and HbAA ghosts. Full spectra from sickle cell haemoglobin homozygote (HbSS)(upper panel) and healthy (HbAA)(lower panel) ghosts, whose partial spectra are shown in Fig. 1. Zoom factors are indicated by the percentages on top of the intensity axis. Red boxes indicate high mannoses. For calculations of percentages high mannose and complex N-glycans, the upper panel of each N-glycan profile was used.

Supplementary Fig. 4

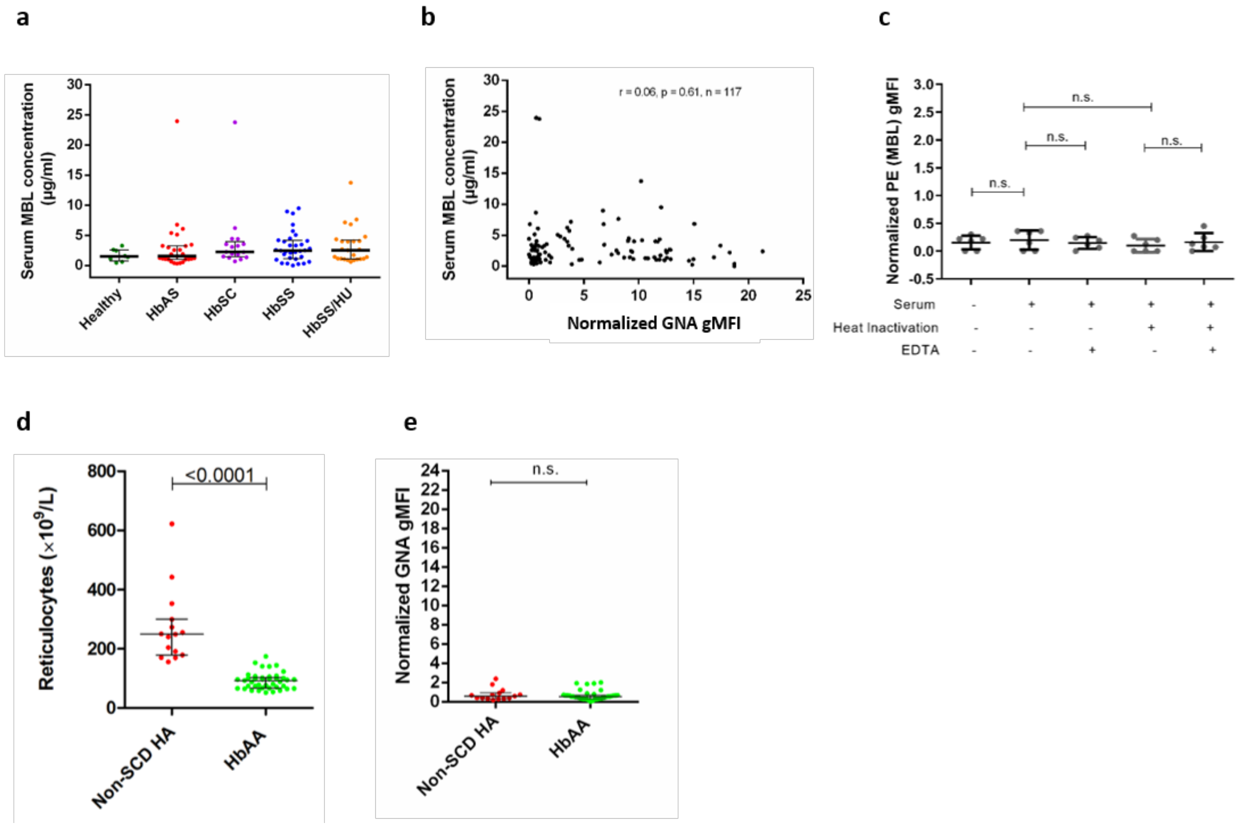

Supplementary Figure 4: Mannose binding lectin correlations and stratification of mannose relationship to anaemia.

Concentrations of serum mannose binding lectin (MBL): (a) split by clinical phenotype (healthy (HbAA)(n=8); sickle cell trait (HbAS)(n=27); heterozygotes for haemoglobins S and C (HbSC)(n=17); homozygotes for sickle cell haemoglobin (HbS)(not receiving hydroxycarbamide treatment, n=32); HbSS/HU (receiving hydroxycarbamide treatment, n=25), (b) correlation with surface *Galanthus nivalis* Agglutinin (GNA) lectin binding, Spearman's rank correlation and (c) lack of direct binding to HbSS RBCs (n=6), 2-tailed Mann-Whitney. Comparison of reticulocyte counts, (2 tailed Mann-Whitney, $p=1.8\times10^{-8}$) (d) or normalized GNA lectin binding geometric mean fluorescence (gMFI)(2 tailed Mann-Whitney) (e) between HbAA and non-sickle cell disease haemolytic anaemias (Non-SCD HA). For d), HbAA (n=15), non-SCD (n=37). For e), HbAA (n=15), non-SCD (n=41). Each data point indicates an independent RBC donor over two or more independent experiments. Data shown as median +/- IQR. 2 tailed Mann-Whitney with exception for b)

Supplementary Fig. 5

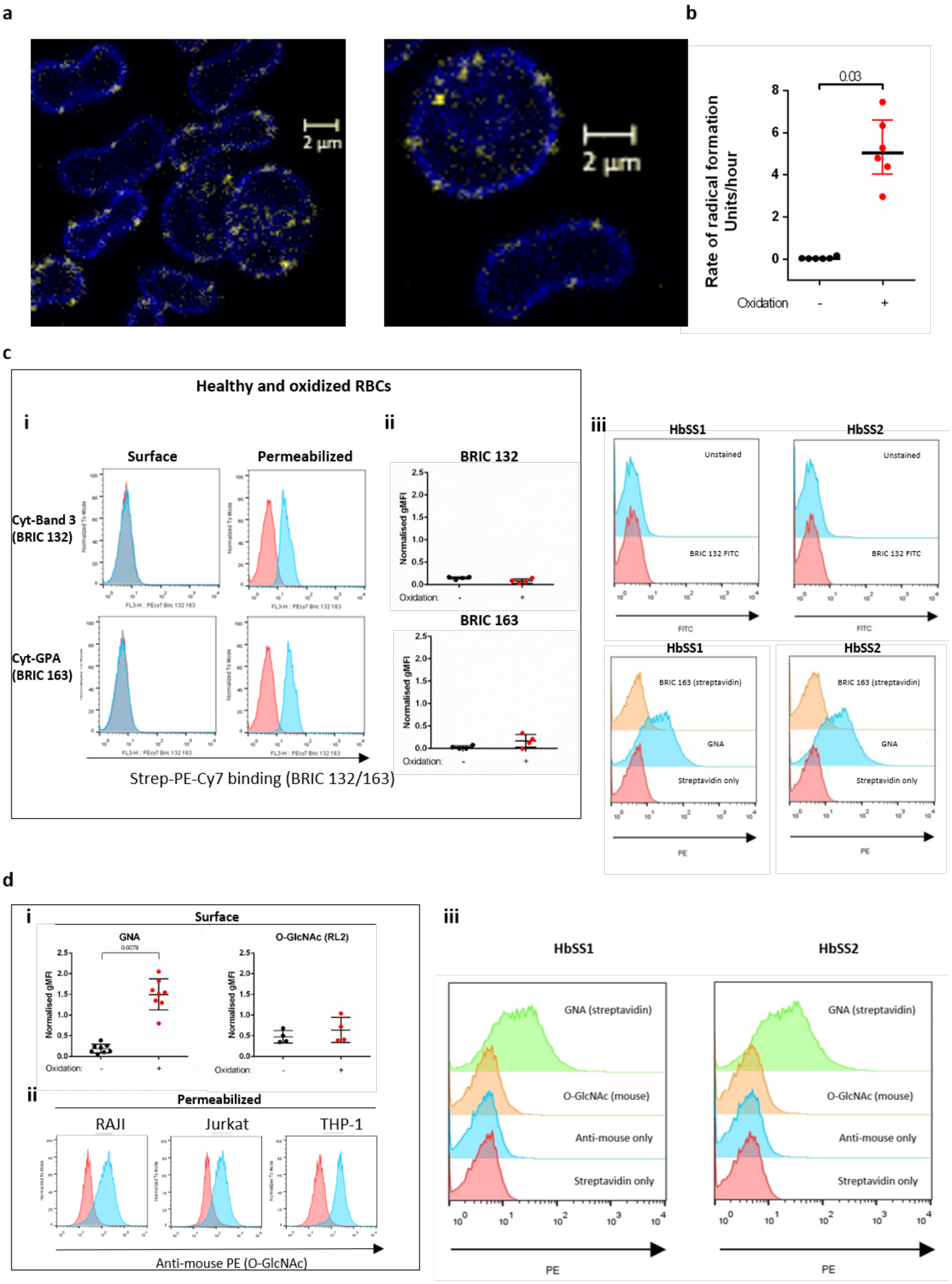

104

Supplementary Figure 5: Oxidation exposes mannose on the surface of RBCs

a) Confocal microscopy of healthy (HbAA) RBCs permeabilized before staining with biotinylated *Galanthus nivalis* Agglutinin (GNA) lectin, streptavidin (yellow) and anti-spectrin (blue).

b) Rate of radical formation calculated from six-hour time course measurement for healthy RBCs with or without oxidation by $CuSO_4$/ascorbic acid during time course. 2 tailed Wilcoxon, n=6 biologically independent donors, representative of two independent experiments.

c) Flow cytometric analysis of healthy, oxidized and sickle cell haemoglobin homozygotes (HbSS) RBCs using BRIC 132 (anti-cytoplasmic band 3) or BRIC 163 (anti-cytoplasmic glycophorin A). Left hand panels: i) histograms for permeabilized versus non-permeabilized binding of HbAA RBCs to BRIC antibodies. ii) BRIC antibody binding to surfaces of oxidized and undamaged HbAA RBCs. Differences not significant by 2 tailed Wilcoxon. n=4 biologically independent donors representative of two independent experiments. Right hand panel (iii): BRIC antibody and GNA lectin binding to RBCs from two HbSS donors, without permeabilization.

d) Flow cytometric analysis of healthy, oxidized and HbSS RBC using O-GlcNAc specific antibody, RL2. i) Normalized GNA lectin (n=8) and RL2 (n=4) binding for undamaged and oxidized RBCs, without permeabilization. Each point represents a biologically independent donor representative of two independent experiments. ii) Histograms for RL2 binding to permeabilized nucleated cells shown. Right hand panel (iii): RL2 antibody and GNA lectin binding to RBCs from two HbSS donors, without permeabilization. All 2 tailed Wilcoxon.

Supplementary Fig. 6

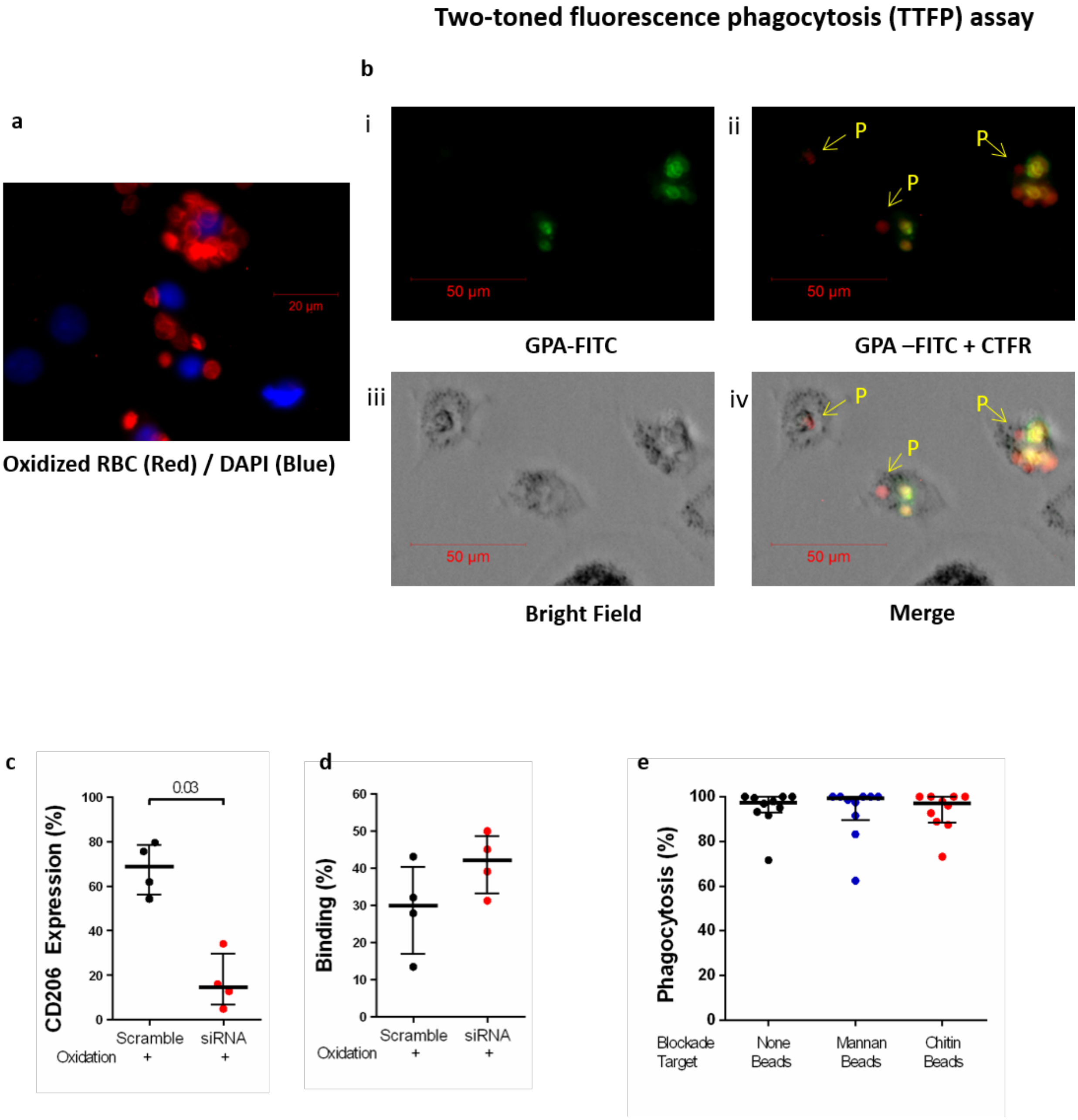

Supplementary Figure 6: Two-toned fluorescence phagocytosis (TTFP) assay

a) Oxidized RBCs stained with Cell Trace Far Red (CTFR, red) are incubated with human monocyte derived macrophages (HMDM) for 3 hours, washed with phosphate buffered saline (PBS), permeabilized and stained for nucleus (DAPI, blue). Immunofluorescence. Scale bar shown.

b) Oxidized RBCs stained with Cell Trace Far Red (CTFR, red) are incubated with HMDM for 3 hours, washed with PBS, and counter-stained prior to immunofluorescence microscopy with anti-GPA-FITC antibody (green) to identify RBCs that are not sequestered inside macrophages: GPA-FITC only (i), GPA-FITC and CTFR (ii), bright field only (iii) and merged (iv). CTFR (red) single positive cells are counted as having been phagocytosed (letter E). Double positive (CTFR and GPA) cells are not counted as having been phagocytosed, but bound to the macrophage cell surface. Scale bar 50 μm scale as shown.

c) Mannose receptor (CD206) expression assessed by microscopy for human monocyte derived macrophages treated with siRNA or scramble control. 2 tailed Mann-Whitney, n=4 biologically independent RBC donors over two independent experiments.

d) Percentage surface binding of sickle cell haemoglobin homozygote (HbSS) RBCs assessed by microscopy for human monocyte derived macrophages treated with siRNA or scramble control. 2 tailed Mann-Whitney, n=4 biologically independent RBC donors over two independent experiments.

e) Quantified phagocytosis of beads by HMDM with and without glycan polymer blockade, applied in the same way and the same concentration as for RBC phagocytosis experiments.

2 tailed Mann-Whitney, n=10 biologically independent PBMC donors for HMDM over three independent experiments.

Supplementary Fig. 7

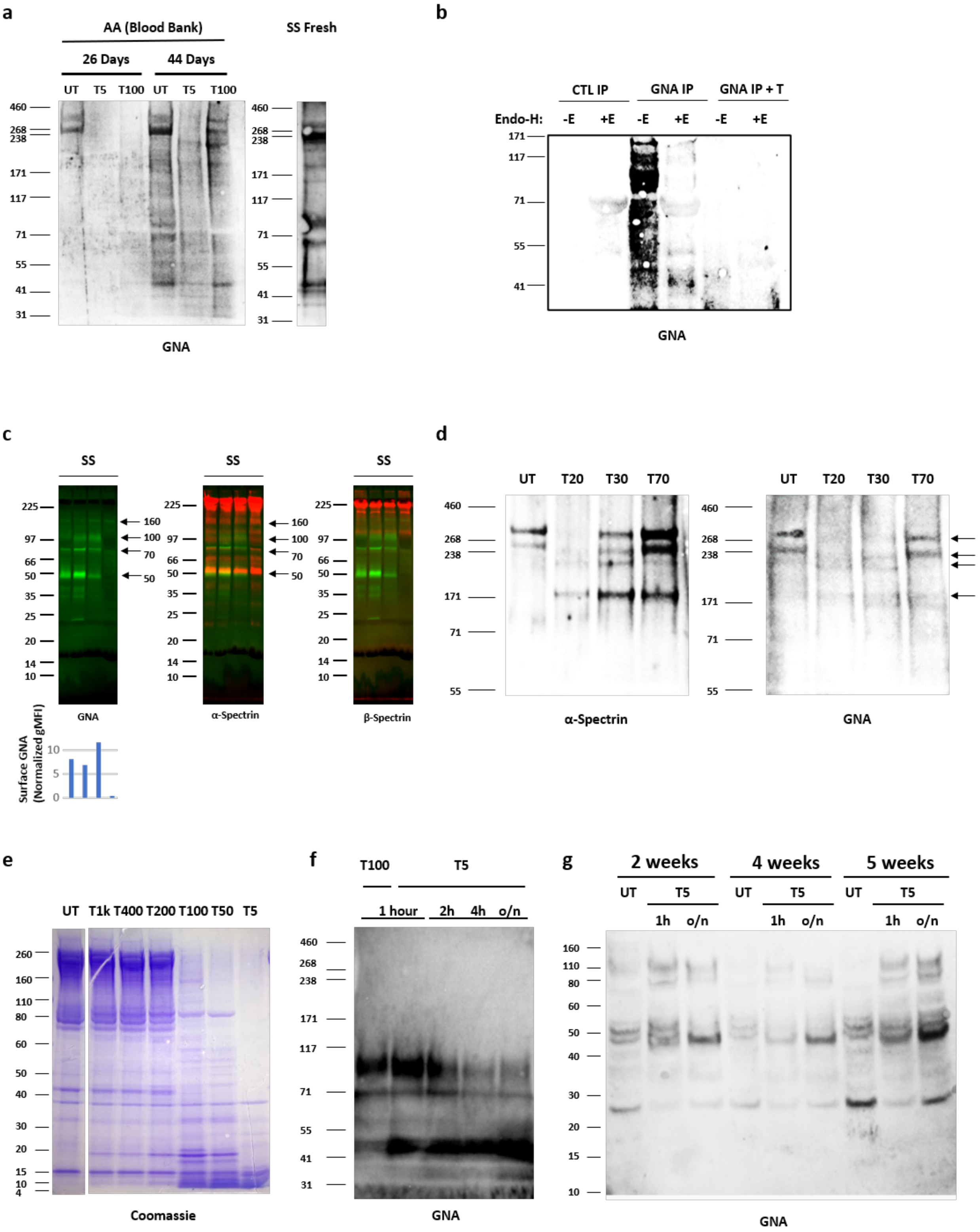

Supplementary Figure 7: High mannose positive fragments derived from aging and proteolysis

a) *Galanthus nivalis* Agglutinin (GNA) lectin western from healthy (HbAA) ghosts isolated from erythrocytes stored from 26 to 44 days. T5 and T100 indicate trypsin digestion of ghosts, where the number indicates the dilution factor of trypsin. Comparison sickle cell haemoglobin homozygote (HbSS) GNA lectin blot is shown, demonstrating correspondence in the positions of the ~160kDa, ~100kDa, ~70kDa and ~50kDa fragments.

b) Stored RBC (>40 days) ghosts are Triton treated and subjected to GNA lectin precipitation or control (no lectin) precipitation. Endo-H (+E) or control digestion (-E) is applied to the eluates. Overnight trypsin treatment is also applied to a subset of GNA immunoprecipitation (IP) elution (GNA IP + T), prior to Endo-H or control digestions.

c) Dual colour western blot from HbSS ghosts using GNA lectin (green) with either α-spectrin or β-spectrin antibodies (both red). Below are corresponding surface FACS GNA lectin staining (normalized geometric mean fluorescence (gMFI)). N.B. The 8-18% gradient gel used here does not yield bright 260kDa GNA lectin binding bands.

d) Partial trypsin digestion of HbAA ghosts (T70 indicates 1:70 ratio of trypsin to sample, etc) then blotted with polyclonal α-spectrin antibody or GNA lectin.

e) Coomassie stain of spectrin released from HbSS ghosts after digestion with trypsin for one hour. Untreated (UT), Tx indicates the dilution factor of trypsin relative to spectrin material.

f) GNA lectin blot of HbSS ghost after prolonged trypsin treatment from 1 hour to overnight (20hours).

g) GNA lectin blot of ghosts made from HbSS erythrocytes aged from 2 to 5 weeks. Untreated (UT), one hour (1h) and overnight (o/n) high concentration trypsin digestions (T5).

Supplementary Fig. 8

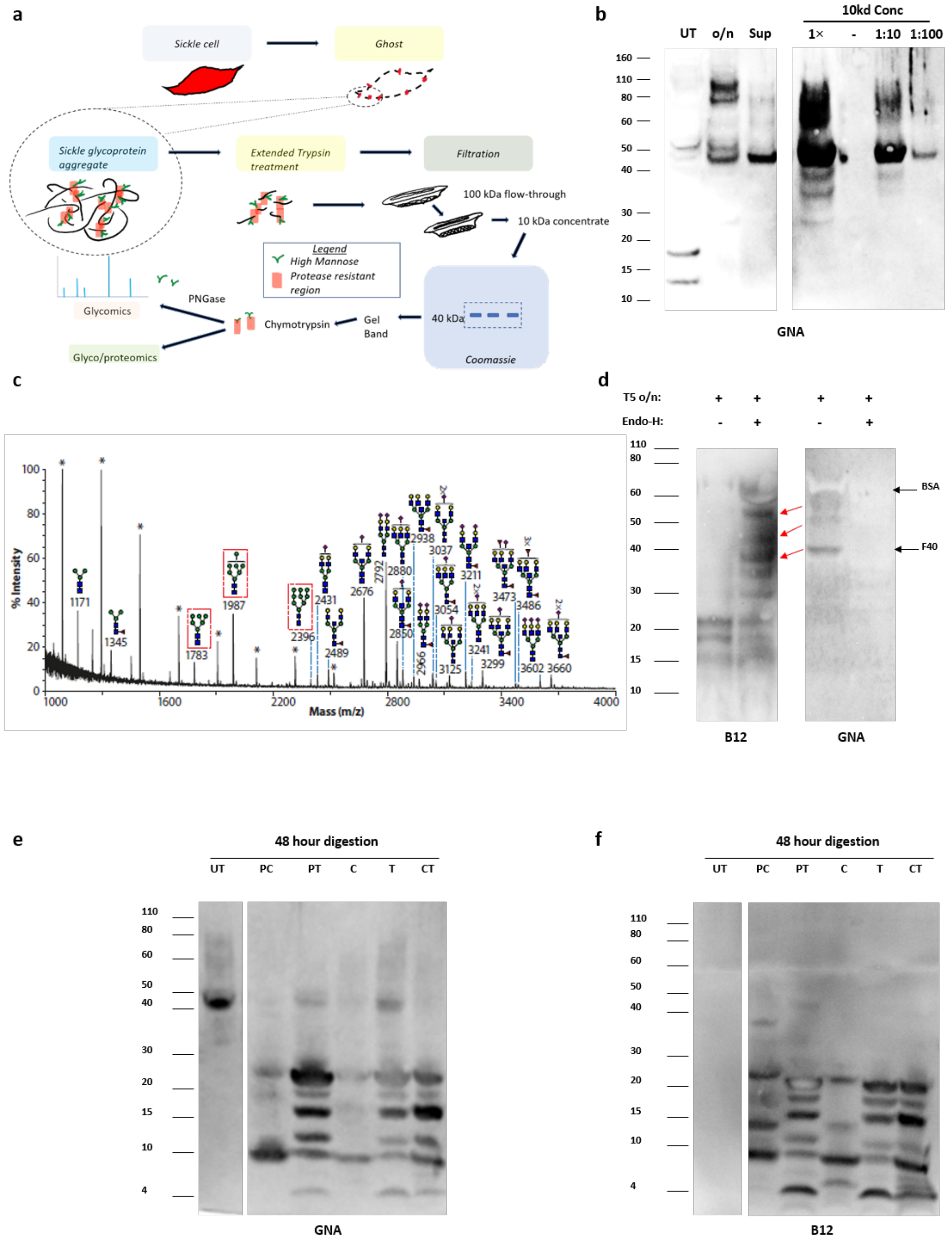

Supplementary Figure 8: Purification and analysis of protease resistant F40 from sickle cells.

a) Cartoon outlining purification process for peptide F40. Sickle cells (top right) are used to make RBC ghosts, which are then subjected to prolonged incubation with trypsin before removing the ghosts by centrifugation. The supernatant is then passed through a 100 kDa filter and concentrated on a 10 kDa filter before running on a polyacrylamide gel. The ~40 kDa band is cut out and digested with chymotrypsin (then PNGase) before analysis by mass spectrometry for glycopeptides (and glycans).

b) *Galanthus nivalis* Agglutinin (GNA) lectin blot of enrichment process of F40 from aged homozygote sickle cell haemoglobin (HbSS) erythrocyte ghosts. Untreated (UT); overnight T5 (o/n); supernatant from heat inactivated o/n sample (Sup). Final product from sequential concentration with 100kDa and 10kDa concentrators is shown as 1×, 1:10 and 1:100 dilution series. See explanatory notes below.

c) Glycomic analysis following PNGase release of N-linked glycans from F40. Red boxes indicate high mannoses. Putative structures based on composition, tandem MS and biosynthetic pathways. All ions are $[M+Na]^+$. Peaks annotated with an asterisk (*) do not correspond to glycan structures. Major structures are annotated for clarity.

d) HbSS 10kDa concentrate (from a) was digested with trypsin (T5) overnight, heat inactivated, then digested with Endo-H or control for 24 hours. GNA lectin and B12 western blots. Red arrows show likely migration of GNA lectin and B12 reactive bands. Black arrows indicate contaminating BSA and position of F40.

e) HbSS 10kDa concentrate (from a) subjected to combinatorial protease digestion in mild denaturing conditions over 48 hours. Western blot with GNA lectin. (UT) untreated, (PC) pepsin 24 hours then chymotrypsin 24 hours, (PT) pepsin 24 hours then trypsin 24 hours,

(C) chymotrypsin 48 hours, (T) trypsin 48 hours, (CT) chymotrypsin 24 hours then trypsin 24 hours, with heat inactivation at 24 hours.

f) As d) but with western blotting using antibody B12.

Purification and properties of F40: F40 from overnight trypsin digestion of HbSS RBC ghosts partitions to the supernatant following heat inactivation (a). We thereby concentrated F40, through sequential 100kDa and 10kDa cut off concentrators, by approximately 50-fold (a). This preparation exhibited remarkably high GNA lectin binding, approximately 100-fold greater than full length spectrin. Sufficient F40 was purified for glycoproteomic analysis. PNGase released glycans including three high mannoses, a tri-mannose structure and other complex glycans (b). The Endo-H sensitive nature of F40 GNA lectin binding restricts the GNA lectin ligands within this pool of structures to the high mannoses: $Man_6GlcNAc_2$, $Man_8GlcNAc_2$ and $Man_9GlcNAc_2$. GNA lectin reactivity to F40 shows partial sensitivity to digestion with chymotrypsin and proteomic analysis of this digest showed the main protein represented was α-spectrin (Fig. 4i). The most abundant peptides came from the N-terminal 370 amino acids, spanning spectrin repeats 1 to 3, with only scattered, low abundance peptides identified from the rest of the molecule and none from β-spectrin. Excluding mass spectrometry incompatible regions from this region yielded 34% coverage across spectrin repeats 1 to 3, with 64% coverage within repeat 3.

Identification of the N-terminal portion of α-spectrin as a major constituent of F40 from mass spectrometry analysis was also supported by western blotting using B12, an N-terminus specific α-spectrin antibody. The antibody does not bind GNA lectin reactive F40 or two higher molecular weight bands directly, but epitopes are revealed by removal of high mannoses using Endo-H (c). Furthermore, three new B12-binding bands migrated at a slightly lower positions

relative to the three GNA lectin binding bands, consistent with glycosidase induced cleavages (c). 48 hour-long digestions with combinations of proteases were able to cleave F40 and, even without Endo-H treatment, unmask B12-binding epitopes (e). The GNA lectin and B12-binding fragments align remarkably well under all treatment combinations (d, e). These data suggest the high mannose decoration and unusual protein structure of F40 limits antibody access and may account for its resistance to proteases.

Supplementary Fig. 9

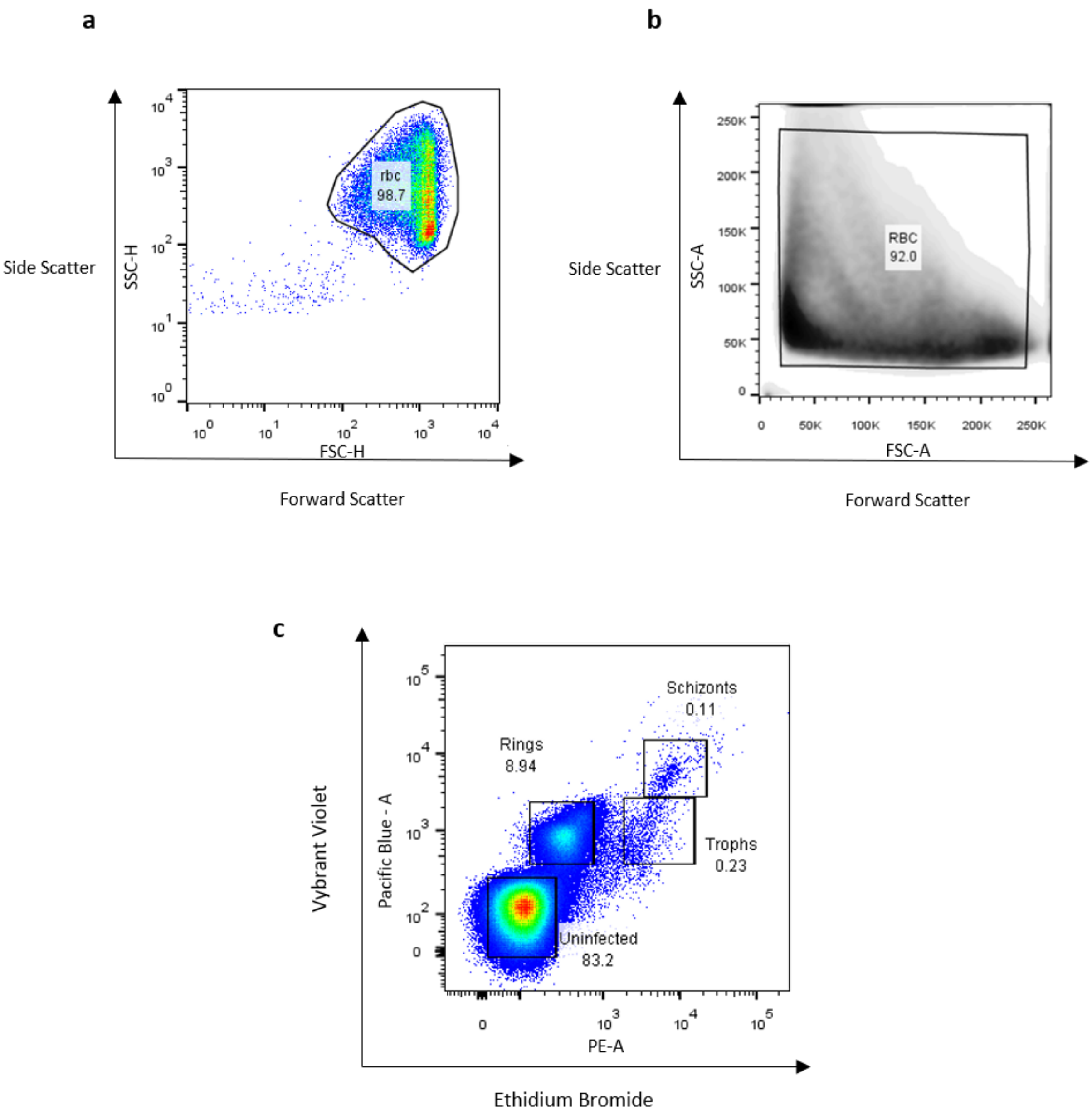

Supplementary Figure 9. Gating strategy for flow cytometric analysis.

a) Example of gating strategy for selecting whole red blood cells used in both whole blood and purified RBC flow cytometry (with the exception of *P. falciparum* infected RBC flow cytometry). Applies to all flow cytometry related data with exception for Fig 5c-d.

b) Example of first gating of whole red blood cells used in analysis of *P. falciparum* infected RBC flow cytometry. Applies to Fig. 5c-d.

c) Example of second gating of *P. falciparum* infected RBCs by maturation stages as defined by ethidium bromide (PE) and Vybrant Violet (Pacific Blue) staining. Applies to Fig. 5c-d.
