# Supplementary material for: Red blood cell mannoses as phagocytic ligands mediating both sickle cell anaemia and malaria resistance
Source: Nat Commun. 2021 Mar 19;12:1792. doi: 10.1038/s41467-021-21814-z (PMC7979802; doi:10.1038/s41467-021-21814-z)
Supplement: Supplementary file 3 — Reporting Summary [file 41467_2021_21814_MOESM3_ESM.pdf]

nature research

Corresponding author(s): Mark Vickers

Last updated by author(s): 2021/02/05

Reporting Summary

Nature Research wishes to improve the reproducibility of the work that we publish. This form provides structure for consistency and transparency in reporting. For further information on Nature Research policies, see our Editorial Policies and the Editorial Policy Checklist.

Statistics

For all statistical analyses, confirm that the following items are present in the figure legend, table legend, main text, or Methods section.

n/a	Confirmed	
☐	☒	The exact sample size (*n*) for each experimental group/condition, given as a discrete number and unit of measurement
☐	☒	A statement on whether measurements were taken from distinct samples or whether the same sample was measured repeatedly
☐	☒	The statistical test(s) used AND whether they are one- or two-sided<br>*Only common tests should be described solely by name; describe more complex techniques in the Methods section.*
☒	☐	A description of all covariates tested
☐	☒	A description of any assumptions or corrections, such as tests of normality and adjustment for multiple comparisons
☐	☒	A full description of the statistical parameters including central tendency (e.g. means) or other basic estimates (e.g. regression coefficient) AND variation (e.g. standard deviation) or associated estimates of uncertainty (e.g. confidence intervals)
☐	☒	For null hypothesis testing, the test statistic (e.g. *F*, *t*, *r*) with confidence intervals, effect sizes, degrees of freedom and *P* value noted<br>*Give P values as exact values whenever suitable.*
☒	☐	For Bayesian analysis, information on the choice of priors and Markov chain Monte Carlo settings
☒	☐	For hierarchical and complex designs, identification of the appropriate level for tests and full reporting of outcomes
☐	☒	Estimates of effect sizes (e.g. Cohen's *d*, Pearson's *r*), indicating how they were calculated

Our web collection on statistics for biologists contains articles on many of the points above.

Software and code

Policy information about availability of computer code

Data collection: *Provide a description of all commercial, open source and custom code used to collect the data in this study, specifying the version used OR state that no software was used.*

Data analysis: *Provide a description of all commercial, open source and custom code used to analyse the data in this study, specifying the version used OR state that no software was used.*

For manuscripts utilizing custom algorithms or software that are central to the research but not yet described in published literature, software must be made available to editors and reviewers. We strongly encourage code deposition in a community repository (e.g. GitHub). See the Nature Research guidelines for submitting code & software for further information.

Data

Policy information about availability of data

All manuscripts must include a data availability statement. This statement should provide the following information, where applicable:

- Accession codes, unique identifiers, or web links for publicly available datasets
- A list of figures that have associated raw data
- A description of any restrictions on data availability

Source data can be accessed at: https://osf.io/fv6h5/?view_only=65091838d5bb4229b509f704a1e0c391 (DOI 10.17605/OSF.IO/FV6H5) (54). The mass spectrometry proteomics data have been deposited to the ProteomeXchange Consortium via the PRIDE (55) partner repository with the dataset identifier PXD024022. The corresponding author will reply to enquiries about unprocessed raw data upon reasonable request.

Field-specific reporting

Please select the one below that is the best fit for your research. If you are not sure, read the appropriate sections before making your selection.

- [x] Life sciences
- [ ] Behavioural & social sciences
- [ ] Ecological, evolutionary & environmental sciences

For a reference copy of the document with all sections, see nature.com/documents/nr-reporting-summary-flat.pdf

Life sciences study design

All studies must disclose on these points even when the disclosure is negative.

Sample size	No formal power calculations were performed.
Data exclusions	Many of these protocols needed development and optimising. Data generated during this process have not been included. However, the phenomena that we report were observed, often to a lesser extent during these optimisations, which also increase our confidence in the data.
Replication	The number of samples analysed for each lectin in the initial survey (Fig. 1) are </=7. However, the important conclusions from Figure 1 were corroborated by analysing over 100 clinical samples (Fig. 2 and Extended Data Fig. 3).<br>The number of individual experiments in each group in Fig 2f are 4-7. However, the data generated using oxidized HbAA RBC in essentially the same experiments are greater (Fig. 3k-n) and corroborate our conclusions.<br>For the experiments performed with P. falciparum, several optimisation runs The number of experiments in the malaria section were originally 5, and reached 'significance', but were extended to 10 to increase statistical confidence.
Randomization	N/A
Blinding	Clinical samples were analysed blindly before the resultant data were correlated with RBC phenotypes. Otherwise formal blinding was not employed in the project.

Reporting for specific materials, systems and methods

We require information from authors about some types of materials, experimental systems and methods used in many studies. Here, indicate whether each material, system or method listed is relevant to your study. If you are not sure if a list item applies to your research, read the appropriate section before selecting a response.

Materials & experimental systems

n/a	Involved in the study	
[ ]	[x]	Antibodies
[x]	[ ]	Eukaryotic cell lines
[x]	[ ]	Palaeontology and archaeology
[x]	[ ]	Animals and other organisms
[x]	[ ]	Human research participants
[x]	[ ]	Clinical data
[x]	[ ]	Dual use research of concern

Methods

n/a	Involved in the study	
[x]	[ ]	ChIP-seq
[ ]	[x]	Flow cytometry
[x]	[ ]	MRI-based neuroimaging

Antibodies

Antibodies used	All antibodies are listed in the methods, with suppliers and codes.
Validation	*Describe the validation of each primary antibody for the species and application, noting any validation statements on the manufacturer's website, relevant citations, antibody profiles in online databases, or data provided in the manuscript.*

Flow Cytometry

Plots

Confirm that:

- [x] The axis labels state the marker and fluorochrome used (e.g. CD4-FITC).
- [x] The axis scales are clearly visible. Include numbers along axes only for bottom left plot of group (a 'group' is an analysis of identical markers).
- [x] All plots are contour plots with outliers or pseudocolor plots.
- [x] A numerical value for number of cells or percentage (with statistics) is provided.

Methodology

Sample preparation	Whole blood samples were obtained from donors. Whole blood samples were used for whole blood flow cytometry assays. Red blood cells were purified (see Materials and Methods) and used for purified red blood cell flow cytometry assays. Purified red blood cells were used for malaria infection experiments (see Materials and Methods).
Instrument	BD FACSCalibur. Supplier: Becton Dickinson; Model: FACSCalibur 4CLR (For RBC flow cytometry); and BD LSR SORP Fortessa. Supplier: Becton Dickinson; Model: 647794E6. (for malaria work only).
Software	BDFACSDiva (Fortessa) and BD CellQuest(TM) Pro (BD).
Cell population abundance	Purity was determined to be greater than 99% RBCs by GPA staining.
Gating strategy	Gating strategies are shown in Extended Data Fig. 9 and discussed in Materials and Methods.

☒ Tick this box to confirm that a figure exemplifying the gating strategy is provided in the Supplementary Information.
